# Recurrent Genomic Gains in Preinvasive Lesions as a Biomarker of Risk for Lung Cancer

**DOI:** 10.1371/journal.pone.0005611

**Published:** 2009-06-09

**Authors:** Pierre P. Massion, Yong Zou, Hasmet Uner, Porntip Kiatsimkul, Holly J. Wolf, Anna E. Baron, Tim Byers, Steinn Jonsson, Stephen Lam, Fred R. Hirsch, York E. Miller, Wilbur A. Franklin, Marileila Varella-Garcia

**Affiliations:** 1 Division of Allergy, Pulmonary and Critical Care Medicine, Vanderbilt Ingram Cancer Center, Veterans Administration Medical Center, Nashville, Tennessee, United States of America; 2 Department of Medicine, University of Colorado Denver, Denver, Colorado, United States of America; 3 Department of Preventive Medicine and Biostatistics, University of Colorado Denver, Denver, Colorado, United States of America; 4 Department of Pathology, University of Colorado Denver, Denver, Colorado, United States of America; 5 Pulmonary Division, Department of Medicine, Denver Veterans Affairs Medical Center Denver, Denver, Colorado, United States of America; 6 Department of Medicine University of Iceland Hospitals, Reykjavik, Iceland; 7 Department of Respiratory Medicine, University of British Columbia, Vancouver, Canada; National Cancer Institute, United States of America

## Abstract

Lung carcinoma development is accompanied by field changes that may have diagnostic significance. We have previously shown the importance of chromosomal aneusomy in lung cancer progression. Here, we tested whether genomic gains in six specific loci, *TP63* on 3q28, *EGFR* on 7p12, *MYC* on 8q24, 5p15.2, and centromeric regions for chromosomes 3 (*CEP3*) and 6 (*CEP6*), may provide further value in the prediction of lung cancer. Bronchial biopsy specimens were obtained by LIFE bronchoscopy from 70 subjects (27 with prevalent lung cancers and 43 individuals without lung cancer). Twenty six biopsies were read as moderate dysplasia, 21 as severe dysplasia and 23 as carcinoma *in situ* (CIS). Four-micron paraffin sections were submitted to a 4-target FISH assay (LAVysion, Abbott Molecular) and reprobed for *TP63* and CEP 3 sequences. Spot counts were obtained in 30–50 nuclei per specimen for each probe. Increased gene copy number in 4 of the 6 probes was associated with increased risk of being diagnosed with lung cancer both in unadjusted analyses (odds ratio = 11, p<0.05) and adjusted for histology grade (odds ratio = 17, p<0.05). The most informative 4 probes were *TP63*, *MYC*, CEP3 and CEP6. The combination of these 4 probes offered a sensitivity of 82% for lung cancer and a specificity of 58%. These results indicate that specific cytogenetic alterations present in preinvasive lung lesions are closely associated with the diagnosis of lung cancer and may therefore have value in assessing lung cancer risk.

## Introduction

Early diagnosis of lung cancer is thought to lead to improved survival. Yet, less then 25% of patients are diagnosed at clinical stage 1 where expected survival is around 70% at 5 years. This survival rate is much higher than overall survival in advanced disease, estimated at 15% at 5 years. Approaches to early diagnosis of lung cancer remain a major challenge. The onset of the disease process is extremely slow (months to years) and no means of evaluating the rate of progression of the disease process are available.

A variety of lung cancer screening techniques have been studied to determine their utility in early stage of disease. These include chest X-ray, sputum cytology and molecular biomarkers in various biological specimens. None of these early detection strategies has been found to cause a reduction in cancer-related mortality. Low-dose spiral computed tomography (CT) may provide an accurate picture of the anatomic extent of early lung carcinoma [1]. Yet, although appealing as an early detection strategy, [2], [3], [4], [5], [6], the results of randomized, controlled studies are not known. In addition, most preinvasive lesions in the central airways will remain undetected by chest CT.

Molecular detection strategies from airway specimens are challenging because of the relatively difficult access, paucity of tissue and lack of molecular changes predictive of cancer. While the molecular biology of lung cancer has been extensively studied, no reliable diagnostic molecular correlates exist [7]. Lung cancer development is characterized by sequential accumulation of epigenetic and genetic aberrations in somatic cells [8]. These aberrations include single nucleotide point mutations, changes in chromosome copy number [9], [10], and specific genomic amplifications or deletions that are implicated in the pathogenesis of lung tumor development and progression through the activation of oncogenes and inactivation of tumor suppressor genes [11], [12], [13], [14], [15], [16].

Because not all preinvasive lesions develop into invasive tumors, it is critical to identify molecular determinants driving to an invasive phenotype [16]. Fluorescence in situ hybridization is emerging as a potentially useful clinical tool for the assessment of diagnosis, prognosis, and response to therapy in lung cancer [17], [18], [19], [20], [21], [22]. Chromosomal aneuploidy has been found closely associated with the diagnosis of lung cancer. Recently we tested gain in copy numbers of two out of four selected DNA targets, taken as a reflection of genomic instability and a marker for risk of lung cancer development [10], [23]. In the present study, we hypothesized that a selected set of cytogenetic alterations in preinvasive lesions may be a better predictor of lung cancer. Therefore, we determined whether the results of the cytogenetic analysis were associated with disease progression in the elected individuals. We selected six DNA targets commonly amplified in lung cancer [12], [14], [15], [24], [25] including two centromeric probes (CEP3 and CEP6) and four probes to areas of frequent genomic amplification, i.e. 3q28 (*TP63*) [11], [13], 5p15.2 (D523 and D5S721 markers) [26], 8q24 (*MYC*) [27], and 7p12 (*EGFR*) [28]. With those validated FISH probes, we performed a quantitative evaluation of nuclear representation of genomic locus copy number in preinvasive lesions and their association with a diagnosis of invasive lung carcinoma.

## Materials and Methods

### Patient population characteristics

The population included 70 subjects recruited at the University of Colorado Cancer Center (UCCC), the British Columbia Cancer Agency (BCCA) and the University of Iceland Hospitals (UIH). This population represents a subgroup of patients previously investigated [23] and includes all subjects from that study with diagnosed moderate dysplasia (4 with lung cancer, 22 controls), severe dysplasia (6 with lung cancer, 15 controls) or carcinoma *in situ* (17 with lung cancer, 6 controls), for whom bronchial sections were available.

The subjects studied were all considered to be at high risk for lung cancer based on a history of at least 30 pack-years of smoking and spirometric evidence of airflow obstruction documented by an FEV1/FVC ration of less than 75% and an FEV1 of less than 70% of predicted. Former smokers were defined as having quit at least one year before the time of enrollment. Pack-years were defined as the average number of packs smoked per day multiplied by the numbers of years smoked. Flexible fiberoptic bronchoscopy was performed with both autofluorescence and white light examination of the airways using either a Xillix LIFE II or OncoLIFE system at the UCCC and BCCA sites; white light examination alone was performed at the UIH. The BCCA cases had been diagnosed in a prospective study of early lung cancer using autofluorescence and white light bronchoscopy or the subjects were enrolled as part of two National Cancer Institute sponsored chemoprevention trials. These included 27 patients with clinical diagnosis of invasive carcinoma (prevalent cases) and 43 subjects who remained free of invasive tumor for at least one year of follow up (controls). Detailed questionnaire data derived from personal interview were available on all study subjects, including demographic characteristics and smoking history. The study was approved by the local Institutional Review Boards of the Vanderbilt University, the University of Colorado Health Sciences Center, the BCCA- University of British Columbia Clinical Research Ethics Board, the National Bioethics Committee of Iceland and the Icelandic Data Processing Commission.

### Histology and Selection of Areas of Interest

All biopsies obtained at bronchoscopy were scored according to the most recent WHO classification [29]. Biopsies with diagnoses ranging from moderate dysplasia to carcinoma *in situ* were selected for FISH analysis. Diagnostic areas within individual biopsies were reviewed and imaged by a pathologist (WAF), who marked the areas of interest to be specifically examined by FISH.

### FISH for CEP3, TP63 (3q28), D523, D5S721 (5p15.2), CEP6, EGFR (7p12), and MYC (8q24)

Four-micron paraffin sections were initially submitted to a 4-target FISH assay (LAVysion, Abbott Molecular, Des Moines, IL) including sequences encompassing the DNA markers D523 and D5S721 at 5p15.2, centromere 6, the *EGFR* gene at 7p12 and the *MYC* gene at 8q24, according to protocol described elsewhere [23]. All sections analyzed by FISH were sequential to the respective H&E section. Individual nuclei were assessed for the number of fluorescent signal corresponding to a copy number of the gene of interest. Individual spot counts are referred as signals separated at least by the size of one fluorescent signal. After the analysis of these 4 genomic regions, the same sections were stripped of their fluorescence signals in 70% formamide solution at 72°C for 10 minutes and then re-probed with a 2-target FISH assay including *TP63* at 3q28 (BAC clones RP11-53D15 and RP11-373I6, digoxigenin-labeled, detected by FITC) and Spectrum Orange-CEP 3 sequences (Abbott Molecular, Des Moines, IL). Immunochemical detection procedures were as described previously [30].

Hybridized slides were examined in fluorescence microscopes equipped with proper interference filters and coupled with a CytoVision Genetic workstation (Applied Imaging). Over the areas of interest marked by the pathologist, spot counts were obtained for 30–50 nuclei per specimen for each probe and representative images captured digitally. Considering that the bronchial sections had truncated nuclei, for each DNA target the specimen was defined as abnormal when the mean copy number per cell was greater than two.

#### Statistical analysis

We focused first on the distributions of demographic factors, such as age, sex, smoking status (current or ex-smoker) and histologic grades. Next, we examined the differences in mean copy number of each FISH marker based on the histology. Multiple comparison tests using bootstrap technique were performed for significance. Third, the associations between FISH markers and cancer status were assessed individually and in multiplicity models after controlling for clinical and biological parameters, such as smoking status and histology grade, using multiple logistic regression. Associations were reported as odds ratios with corresponding 95% confidence intervals (CI). A mean number of ≤2 copies per nucleus was considered as the FISH reference value. Finally, we used ROC analysis (c-statistics) to investigate the contributions of marker groups in combined models to differentiate cases and controls. All analyses were carried out in Statistical Analysis Software (Version 9.1, SAS Institute Inc, Cary NC). The comparisons of areas under the ROC curves between models were examined using the “ROC Macro” SAS tool [31], [32].

## Results

Patient clinical information and pathological characteristics of the lesions are summarized in Table 1. There was no statistical difference for age, gender, study center and current smoking status between cases and controls. However, the average smoking intensity was greater among the cases (pack year (PKY) mean = 80.1, SD = 46.1) compared with the controls (mean PKY = 56.6, SD = 24.3). Similarly, the distribution of preinvasive lesions was skewed towards higher grades in patients with cancer.

**Table 1:** Distribution of lung cancer cases and controls according to demographic and histology variables.

Characteristics		Individuals with cancer (Cases, N = 27)	Individuals without cancer (Controls, N = 43)	*Chi-square p value*
Gender	males	85%	79%	*0.75*
	females	15%	21%	
Age	30–59	33%	42%	*0.33*
	60–69	30%	37%	
	70+	37%	21%	
Study center	BCCC	15%	21%	*0.34*
	Iceland	18%	7%	
	Colorado	67%	72%	
Smoking pack-years	<50	37%	56%	*0.03*
	50–74	15%	26%	
	75+	41%	14%	
	unknown	7%	4%	
Current smoking	yes	70%	58%	*0.32*
	no	26%	37%	
	unknown	4%	5%	
Histologic grade	moderate dysplasia, n = 26	15%	51%	*<0.001*
	severe dysplasia, n = 21	22%	35%	
	carcinoma in situ, n = 23	63%	14%	

**Figure 1:**
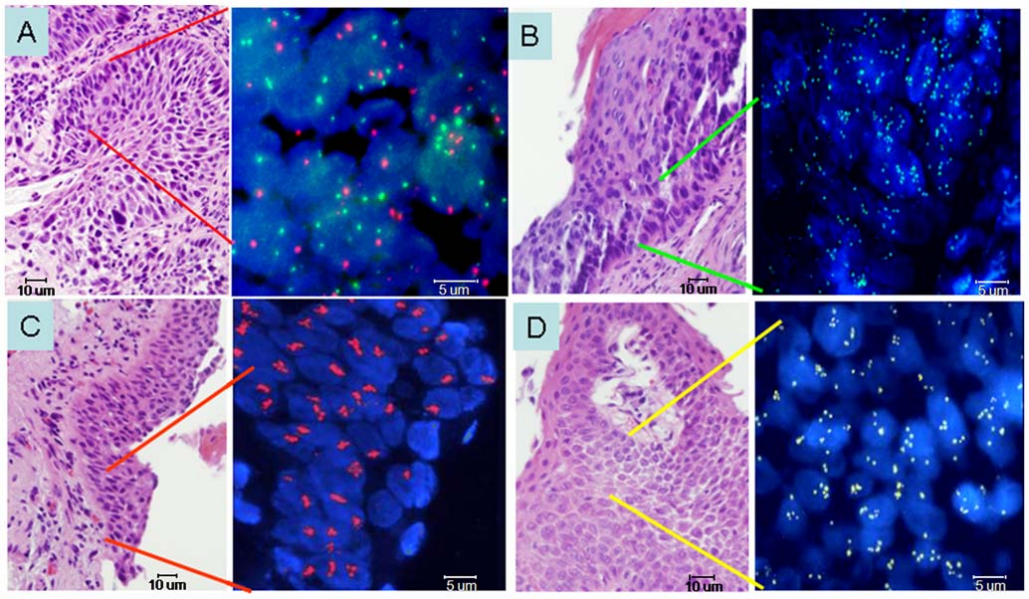
Dual color fluorescence in situ hybridization of *TP63* gene (3q28, green spots) and a CEP3 probe (red spots) on the centromeric region of the same chromosome on a carcinoma *in situ* (CIS) of the lung. Interphase nuclei stained in blue with DAPI show increased copy number of *TP63* in CIS (A). Segmented images of 4-color hybridization assays with the LAVysion probe in severe dysplasia lesions, showing high copy numbers of the 5p15.2 genomic region (green spots) (B); *EGFR* gene amplification (clustered red spots) (C); and high copy numbers of *MYC* (yellow spots) (D). All sections analyzed by FISH were sequential to the H&E section presented.

The mean copy number per cell of selected genomic candidate biomarkers is reported by case and control status for different grades of preinvasive lesions in Table 2, showing a tendency of increasing in copy number among samples as the histology grade advances. While the association with histology grade was significant for *TP63*, *MYC*, CEP6, and CEP3 (p<0.01), it was not for *EGFR* and 5p15.2. When these relationships were analyzed according to the proportion of cells that contained more than two copies of each individual marker, we observed associations limited to these same 4 markers. Interestingly, amplification of sequences was detected in a total of 8 specimens, for four of the tested targets: *TP63*, 5p15.2, *EGFR* and *MYC*. Amplification was represented by small to medium size clusters of signals (EGFR) or by more than 50% of cells carrying more than 5 copies of the signals (MYC, 5p15.2 and TP63). Representative images are presented in Figure 1.

**Table 2:** Mean copy number per cell of selected FISH markers by histology grade in bronchial sections of 70 subjects.

Study Subjects	Histologic grade	N	FISH marker (mean copy number and SD per cell)					
			CEP3	TP63	5p15	CEP6	EGFR	MYC
Cases	MD	4	1.84(0.05)	1.85(0.08)	3.45(1.77)	1.71(0.18)	2.76(1.17)	2.27(0.51)
	SD	6	1.86(0.12)	1.92(0.21)	2.94(1.44)	1.99(0.43)	2.84(1.27)	2.59(1.21)
	CIS	17	2.21(0.7)	3.14(1.11)	2.82(0.91)	2.29(0.6)	3.01(2.05)	2.95(0.63)
	*P value* *		*0.56*	*0.04*	*0.98*	*0.25*	*1.00*	*0.53*
Controls	MD	22	1.82(0.11)	1.88(0.21)	2.94(1.56)	1.83(0.23)	2.51(1.06)	2.03(0.56)
	SD	15	1.93(0.23)	2.12(0.58)	2.4(1.04)	1.75(0.37)	2.59(1.69)	2.03(0.6)
	CIS	6	2.02(0.25)	2.74(0.84)	2.21(0.88)	2.08(0.77)	3.25(3.08)	2.3(0.9)
	*P value* *		*0.92*	*0.12*	*0.88*	*0.90*	*0.98*	*0.98*
All	MD	26	1.82(0.1)	1.88(0.19)	3.02(1.57)	1.81(0.22)	2.54(1.05)	2.07(0.55)
	SD	21	1.91(0.2)	2.06(0.5)	2.55(1.16)	1.82(0.39)	2.66(1.56)	2.19(0.83)
	CIS	23	2.16(0.62)	3.04(1.04)	2.66(0.92)	2.23(0.64)	3.07(2.29)	2.78(0.75)
	*P value* *		*0.01*	*<0.0001*	*0.86*	*0.006*	*0.80*	*0.003*

* ANOVA for differences in mean copy number per cell across the three histology grades.

The percentage of lesions abnormal for each FISH marker (according to the case or control status), with abnormality defined as mean copy number per cell greater than two is presented in Table 3. The presence of a malignancy in the airways was only moderately associated with the rate of copy number abnormalities except for *MYC*, which was more frequently amplified in preinvasive lesions of patients with lung cancer.

**Table 3:** Percentage of lesions with abnormal FISH copy numbers according to case or control status (70 subjects).

Study Subjects	Mean copy number per cell	FISH marker					
		CEP3	*TP63*	5p15	CEP6	*EGFR*	*MYC*
Cases (n-27)	≤2 copies	67%	48%	33%	63%	30%	26%
	>2 copies	33%	52%	67%	37%	70%	74%
Controls (n = 43)	≤2 copies	84%	72%	53%	84%	53%	77%
	>2 copies	16%	28%	47%	16%	47%	23%
Unadjusted	OR	2.57	2.78	2.3	3.02	2.73	9.42
	(95% CI)	(0.82–8.02)	(1.01–7.61)	(0.84–6.24)	(0.98–9.31)	(0.98–7.57)	(3.09–28.72)
Adjusted*	OR	3.2	4.17	2.66	3.55	3.08	13.08
	(95% CI)	(0.72–14.11)	(0.91–19.00)	(0.90–7.93)	(1.06–11.86)	(1.01–9.33)	(3.45–49.48)

* Adjusted by multiple logistic regression for gender, age, center, and current smoking status.

Because access to normal bronchial epithelium from the same individuals was not possible, moderate dysplasia was used as the reference baseline to measure the association between copy number abnormalities and lung cancer status. As shown in Table 4, the odds of having lung cancer given that a preinvasive lesion had gain for genomic regions increased from 4.23 (1.21–14.8, 95%CI) when 1 or 2 markers of 4 (CEP3, T*TP63*, CEP6, *MYC*) were abnormal to 11 (2.63–45.9, 95%CI) when 3 or 4 markers showed elevated copy numbers. Further adjustment for age, gender, center, current smoking status and histology grade of the lesion increased the odds to 17.

**Table 4:** Association between disease status and abnormal FISH marker (>2 copies per cell) for the top 4 markers (CEP3, *TP63*, CEP6, *MYC*).

Number of abnormal FISH markers among the top 4	Lung cancer cases	Controls	Unadjusted OR (95% CI)	Adjusted OR* (95% CI)
0	18%	58%	1.00 (reference)	1.00 (reference)
1–2	41%	30%	4.23 (1.21–14.8)	4.45 (0.84–23.6)
3–4	41%	12%	10.99 (2.63–45.9)	16.97 (1.47–195.0)

* Adjusted by multiple logistic regression for gender, age, center, current smoking status and histologic grade.

**Figure 2:**
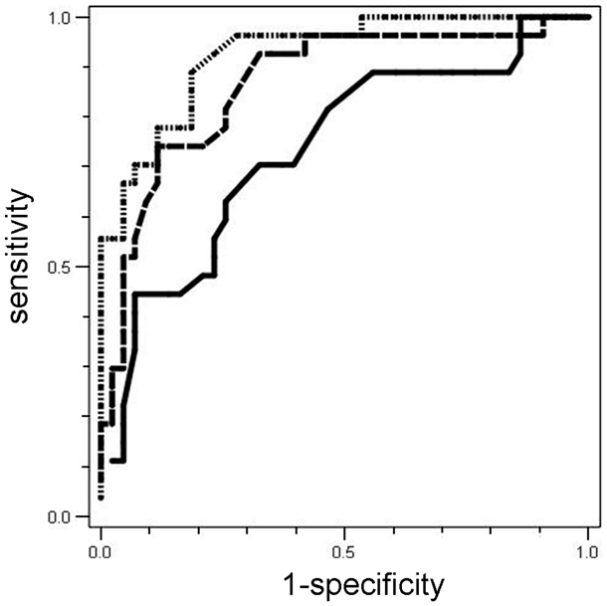
Receiver operating characteristic curves describing the diagnostic prediction accuracy of three models, demographics alone (plain line, area under the curve (AUC) 73.4%), in combination with cytology (dashed line, AUC 86.6%) or demographics in combination with cytology and 4 FISH biomarker candidates (dotted line, AUC 92.6%). The demographic information represents gender, age, pack years smoking history, and smoking status.

When assessed as a candidate biomarker signature predictive of lung cancer, the sensitivity of presence of abnormality in those 4 markers was 82% and specificity was 58%. The receiver operating characteristic (ROC) curves shown in Figure 2 demonstrate the added value of histology and epidemiological information, ultimately achieving an area under the curve of 92.6%. The demographic information represents gender, age, pack years of smoking history, and smoking status. The differences between the curves were significant between demographics vs. demographics and cytology (p = 0.02) or vs. demographics, cytology and 4 FISH biomarkers (p = 0.002). Although showing a trend, the difference was not significant between demographics and cytology vs. demographics, histology and 4 FISH biomarkers (p = 0.11).

## Discussion

Molecular approaches for the early detection for lung cancer have been targeting the blood, the sputum, the exhaled breath and bronchial biopsies [7], [33]. Preinvasive bronchial lesions are well established markers of risk and yet the histological grade does not necessarily predict the outcome [16], [34], [35]. Our goal was to determine whether specific genomic alterations in preinvasive lesions may be predictive of having a lung cancer in high-risk individuals. While genomic instability was addressed previously based on a quantitative analysis [23], a more refined molecular signature is expected to better associate with the diagnosis of lung cancer. Our results indicate that specific cytogenetic alterations present in preinvasive lung lesions such as amplification or over-representation of the *TP63* and *MYC* genes are highly associated with the diagnosis of lung cancer and therefore suggest a role of those markers in assessing lung cancer risk. Unlike p53, *TP63* is rarely mutated in lung cancer but a significant fraction of tumor and premalignant lesions are amplified for both *TP63* and *MYC* genes.

Although chromosomal alterations have been linked to most solid tumors and serve as a hallmark of human cancer [36], they are becoming increasingly complicated; i.e., major patterns are being diluted by many variants [10], [37], [38]. Genomic alterations in the airway epithelium occur both stochastically and later in a clonal manner. Clonal (identical alteration in 2 or more cells) and non-clonal alterations associated with smoking history participate in the tumor initiation process. Some alterations may indicate risk better than others. Thus far, the majority of these alterations have been considered consequences as opposed to causes in the lung cancer development. Some of these low level aberrations have been called random noise but may reflect the measure of true instability. Non-clonal chromosomal alterations (NCCAs) such as defective mitotic figures, chromosomal fragmentation, missegregation or non-recurrent genomic alterations indicate a dynamic process leading to instability [39]. In this study, we found that the high frequency of non-coding centromeric alterations (CEP3 and CEP 6) was independently associated with a diagnosis of lung cancer (Table 2), and therefore although not specifically linked to tumorigenesis, it is probably part of the genomic instability coming along with the disease process.

In contrast, specific genomic amplifications or deletions [12], [15], [40] have been implicated in the pathogenesis of tumor development in part through the activation of oncogenes [41] and inactivation of tumor suppressor genes. Some genomic signatures seem to persist after tumor development, throughout their progression [42] and their histology differentiation. Preinvasive lesions have shown copy number alterations for several chromosomal regions including 3q28, 5p15 and 8q24 [10], [13], [23], [43], [44] and these alterations were also found. Genomic imbalances have been extensively investigated in invasive NSCLCs using CGH or SNP array methodology and numerous loci were found commonly amplified or over-represented in either or both squamous and adenocarcinoma of the lung [14], [15], [24], [25], [45], [46], [47], [48], [49]. If some of them are demonstrated to be early events, this may improve the test performance in the context of diagnosis of lung cancer.

We hypothesized that genomic alterations in *TP63*, 5p15.2, *EGFR*, *MYC*, CEP 6 and CEP 3, may provide a measure of risk assessment. Increased copy number (over-representation or amplification) is technically more reliable to detect with fewer false positives than genomic loss, mainly when using FISH assay in sectioned specimens exhibiting nuclear truncation, thus we have focused on loci that were involved in genomic gain. The cut off for “normal” copy number gain was set at ≤2 copies per nucleus based on the fact that the normal disomic cells have two copies of each genomic target and these cells were sectioned at 4 µm, which generated truncated nuclei. Although this value may not be the optimal cut off to use, it is conservative and assures that samples classified as exhibiting genomic gain are actually abnormal.

*TP63* is an appealing target located at the tip of one of the most prevalent region of amplification in lung cancer. *TP63* is a homologue of p53, which plays a role in development and oncogenesis by regulating proliferation and differentiation. Interest in *TP63* stems from this “two genes in one” concept with agonist and antagonist properties that may be involved in tumor development [50]. *TP63* is a complex gene that has multiple transcriptional isoforms, some of which are tumor suppressors (the *TP63* isoforms), while the others are oncogenes (Δ*TP63*; dN*TP63*) [11].The TA*TP63* isoforms can bind to DNA through p53-responsive elements and therefore “p53-like”. The ΔN*TP63* exerts dominant negative effects over p53 and is proposed as oncogenic. We found that there is an early and frequent genomic amplification of *TP63* in the development of squamous carcinoma of the lung and that patients with NSCLC showing amplification and overexpression of *TP63* have prolonged survival [13]. The ΔΝ*TP63*α splice variant is the most commonly expressed isoform in squamous epithelia [11], [13]. The oncogenic activity of the *TP63* isoforms may explain why we see the amplification and overexpression of this protein. *MYC* is also an important oncogene in lung cancer. It is expressed in a large number of NSCLCs [51]. Gene amplification at 8q24 and resultant increased expression of *MYC* is a common occurrence in carcinomas [48], [52], [53]. It leads to increased formation of the *MYC*:Max heterodimer transcription factors that alter gene expression in large part by recruiting histone-modifying enzymes [47].

Our data suggest that the changes observed for 5p15 and *EGF R* were less predictive of cancer, but these changes seemed to happen earlier in the dysplastic process, at least in smokers. Among 20 bronchial specimens with normal histology from never smokers, none have elevated *EGFR* or 5p15 copy number. Average copy number for these 20 specimens were 1.77 (STD 0.53) for *EGFR* and 1.73 for 5p15 [10]. These observations are also consistent with the observation of frequent *EGFR* mutations (24–43%) found in the airway epithelium in the vicinity of tumors [54], [55], and with the data showing frequent early events on 5p in squamous differentiation of lung cancer [55], [56].

Our design included controls who did not present with lung cancer for at least 12 months after endobronchial biopsy. Since genomic instability occurs not only among cases but also among controls, some of these high-risk controls may eventually develop lung cancer later and our cross sectional study design does not address this risk. Other limitations of the study include the nature of the tissues examined, a relative small sample size (although the study of 70 fully annotated high grade preinvasive lesions required three centers and is one of the largest reported to date), and the inability to study these samples' progression over time.

The use of bronchial biopsies for assessment of lung cancer risk is unlikely to be of optimal clinical use, although it may be useful to predict future cancer in subjects who happen to undergo a biopsy showing high grade bronchial preneoplasia. This type of molecular analysis may have to move to surrogate tissue in the airways including the histologically normal airway epithelium. The small size of the preinvasive lesions and the potential therapeutic effect of biopsies make the evaluation of the progression rate of the aberrations in these tissues rather challenging. Cross-sectional studies allow the investigation of the association between alterations and disease state. Yet, to prove clinical utility, the candidate biomarkers will require further validation in prospective cohort studies.

The accuracy of our cytogenetic signature may be improved in different ways. Genome wide copy number alterations of invasive and preinvasive lesions may allow the selection of regions more specifically associated with the diagnosis of lung cancer. Increasing the number of targets studied in small tissue samples is challenging but newer technologies may help reach this goal. Ultimately, refining a genomic signature observed in preinvasive lesions that predicts who is likely to develop lung cancer would be very informative. In this context, repeated measurement of such alterations and the rate of its accumulation may be particularly valuable in predicting the likelihood of developing lung cancer.

In this study we took advantage of advances in lung cancer molecular genetics and demonstrated that there is a strong association between targeted genomic alterations in preinvasive bronchial lesions and the diagnosis of lung cancer. These alterations can be reliably assessed by FISH, and may represent a method to measure the risk of developing lung cancer. The predictive value of these alterations deserves further evaluation in the airway epithelium of high risk individuals in longitudinal studies.

## References

[1] Kaneko M, Eguchi K, Ohmatsu H, Kakinuma R, Naruke T (1996). Peripheral lung cancer: screening and detection with low-dose spiral CT versus radiography.. Radiology.

[2] Henschke CI, Yankelevitz DF, Libby DM, Pasmantier MW, Smith JP (2006). Survival of patients with stage I lung cancer detected on CT screening.. N Engl J Med.

[3] Bach PB, Jett JR, Pastorino U, Tockman MS, Swensen SJ (2007). Computed tomography screening and lung cancer outcomes.. Jama.

[4] Mulshine JL, Sullivan DC (2005). Clinical practice. Lung cancer screening.. N Engl J Med.

[5] Sobue T, Moriyama N, Kaneko M, Kusumoto M, Kobayashi T (2002). Screening for lung cancer with low-dose helical computed tomography: anti-lung cancer association project.. J Clin Oncol.

[6] Swensen SJ, Jett JR, Sloan JA, Midthun DE, Hartman TE (2002). Screening for lung cancer with low-dose spiral computed tomography.. Am J Respir Crit Care Med.

[7] Wardwell NR, Massion PP (2005). Novel strategies for the early detection and prevention of lung cancer.. Semin Oncol.

[8] Minna JD, Roth JA, Gazdar AF (2002). Focus on lung cancer.. Cancer Cell.

[9] Testa JR, Siegfried JM, Liu Z, Hunt JD, Feder MM (1994). Cytogenetic analysis of 63 non-small cell lung carcinomas: recurrent chromosome alterations amid frequent and widespread genomic upheaval.. Genes Chromosomes Cancer.

[10] Varella-Garcia M, Chen L, Powell RL, Hirsch FR, Kennedy TC (2007). Spectral karyotyping detects chromosome damage in bronchial cells of smokers and patients with cancer.. Am J Respir Crit Care Med.

[11] Hibi K, Trink B, Patturajan M, Westra WH, Caballero OL (2000). AIS is an oncogene amplified in squamous cell carcinoma.. Proc Natl Acad Sci U S A.

[12] Massion PP, Kuo WL, Stokoe D, Olshen AB, Treseler PA (2002). Genomic copy number analysis of non-small cell lung cancer using array comparative genomic hybridization: implications of the phosphatidylinositol 3-kinase pathway.. Cancer Res.

[13] Massion PP, Taflan PM, Jamshedur Rahman SM, Yildiz P, Shyr Y (2003). Significance of p63 Amplification and Overexpression in Lung Cancer Development and Prognosis.. Cancer Res.

[14] Weir BA, Woo MS, Getz G, Perner S, Ding L (2007). Characterizing the cancer genome in lung adenocarcinoma.. Nature.

[15] Zhao X, Weir BA, LaFramboise T, Lin M, Beroukhim R (2005). Homozygous deletions and chromosome amplifications in human lung carcinomas revealed by single nucleotide polymorphism array analysis.. Cancer Res.

[16] Salaun M, Sesboue R, Moreno-Swirc S, Metayer J, Bota S (2008). Molecular predictive factors for progression of high-grade preinvasive bronchial lesions.. Am J Respir Crit Care Med.

[17] Varella-Garcia M (2003). Molecular cytogenetics in solid tumors: laboratorial tool for diagnosis, prognosis, and therapy.. Oncologist.

[18] Sokolova IA, Bubendorf L, O'Hare A, Legator MS, Jacobson KK (2002). A fluorescence in situ hybridization-based assay for improved detection of lung cancer cells in bronchial washing specimens.. Cancer.

[19] Halling KC, Rickman OB, Kipp BR, Harwood AR, Doerr CH (2006). A comparison of cytology and fluorescence in situ hybridization for the detection of lung cancer in bronchoscopic specimens.. Chest.

[20] Bubendorf L, Muller P, Joos L, Grilli B, Vogel S (2005). Multitarget FISH analysis in the diagnosis of lung cancer.. Am J Clin Pathol.

[21] Varella-Garcia M, Mitsudomi T, Yatabe Y, Kosaka T, Nakajima E (2009). EGFR and HER2 genomic gain in recurrent non-small cell lung cancer after surgery: impact on outcome to treatment with gefitinib and association with EGFR and KRAS mutations in a Japanese cohort.. J Thorac Oncol.

[22] Hirsch FR, Varella-Garcia M, Dziadziuszko R, Xiao Y, Gajapathy S (2008). Fluorescence in situ hybridization subgroup analysis of TRIBUTE, a phase III trial of erlotinib plus carboplatin and paclitaxel in non-small cell lung cancer.. Clin Cancer Res.

[23] Jonsson S, Varella-Garcia M, Miller YE, Wolf HJ, Byers T (2008). Chromosomal aneusomy in bronchial high-grade lesions is associated with invasive lung cancer.. Am J Respir Crit Care Med.

[24] Dehan E, Ben-Dor A, Liao W, Lipson D, Frimer H (2007). Chromosomal aberrations and gene expression profiles in non-small cell lung cancer.. Lung Cancer.

[25] Tonon G, Wong KK, Maulik G, Brennan C, Feng B (2005). High-resolution genomic profiles of human lung cancer.. Proc Natl Acad Sci U S A.

[26] Kim NW, Piatyszek MA, Prowse KR, Harley CB, West MD (1994). Specific association of human telomerase activity with immortal cells and cancer.. Science.

[27] Nau MM, Brooks BJ, Carney DN, Gazdar AF, Battey JF (1986). Human small-cell lung cancers show amplification and expression of the N-myc gene.. Proc Natl Acad Sci U S A.

[28] Gazdar AF, Shigematsu H, Herz J, Minna JD (2004). Mutations and addiction to EGFR: the Achilles ‘heal’ of lung cancers?. Trends Mol Med.

[29] Franklin W, Wistuba I, Geisinger K, Lam S, Hirsch F, Travis WDBE, Müller-Hermerlink, Harris CC (2004). Squamous dysplasia and carcinoma in situ.. Tumours of the Lung, Pleura, Thymus and Heart.

[30] Massion P, Gray J, Galas SMaD (2002). Molecular Cytogenetic Explorations of Human Genome.. Genomic Technologies: Present and Future Evolution of Genomic Technologies.

[31] DeLong ER, DeLong DM, Clarke-Pearson DL (1988). Comparing the areas under two or more correlated receiver operating characteristic curves: a nonparametric approach.. Biometrics.

[32] Gonen Mithat (2007). Analyzing Receiver Operating Characteristic Curves with SAS..

[33] Rahman SM, Shyr Y, Yildiz PB, Gonzalez AL, Li H (2005). Proteomic patterns of preinvasive bronchial lesions.. Am J Respir Crit Care Med.

[34] Auerbach O, Stout AP, Hammond EC, Grafinkel L (1961). Changes in bronchial epithelum in reation to cigraette smoking and in relation to lung cancer.. N Engl J Med.

[35] Bota S, Auliac JB, Paris C, Metayer J, Sesboue R (2001). Follow-up of bronchial precancerous lesions and carcinoma in situ using fluorescence endoscopy.. Am J Respir Crit Care Med.

[36] Albertson DG, Collins C, McCormick F, Gray JW (2003). Chromosome aberrations in solid tumors.. Nat Genet.

[37] Ried T, Liyanage M, du Manoir S, Heselmeyer K, Auer G (1997). Tumor cytogenetics revisited: comparative genomic hybridization and spectral karyotyping.. J Mol Med.

[38] Luk C, Tsao MS, Bayani J, Shepherd F, Squire JA (2001). Molecular cytogenetic analysis of non-small cell lung carcinoma by spectral karyotyping and comparative genomic hybridization.. Cancer Genet Cytogenet.

[39] Ye CJ, Liu G, Bremer SW, Heng HH (2007). The dynamics of cancer chromosomes and genomes.. Cytogenet Genome Res.

[40] Balsara BR, Sonoda G, du Manoir S, Siegfried JM, Gabrielson E (1997). Comparative genomic hybridization analysis detects frequent, often high- level, overrepresentation of DNA sequences at 3q, 5p, 7p, and 8q in human non-small cell lung carcinomas.. Cancer Res.

[41] Lockwood WW, Chari R, Coe BP, Girard L, Macaulay C (2008). DNA amplification is a ubiquitous mechanism of oncogene activation in lung and other cancers.. Oncogene.

[42] Wistuba (2007). Genetics of preneoplasia: lessons from lung cancer.. Curr Mol Med.

[43] Garnis C, Coe BP, Ishkanian A, Zhang L, Rosin MP (2004). Novel regions of amplification on 8q distinct from the MYC locus and frequently altered in oral dysplasia and cancer.. Genes Chromosomes Cancer.

[44] Garnis C, Buys TP, Lam WL (2004). Genetic alteration and gene expression modulation during cancer progression.. Mol Cancer.

[45] Choi YW, Choi JS, Zheng LT, Lim YJ, Yoon HK (2007). Comparative genomic hybridization array analysis and real time PCR reveals genomic alterations in squamous cell carcinomas of the lung.. Lung Cancer.

[46] Garnis C, Lockwood WW, Vucic E, Ge Y, Girard L (2006). High resolution analysis of non-small cell lung cancer cell lines by whole genome tiling path array CGH.. Int J Cancer.

[47] Kendall J, Liu Q, Bakleh A, Krasnitz A, Nguyen KC (2007). Oncogenic cooperation and coamplification of developmental transcription factor genes in lung cancer.. Proc Natl Acad Sci U S A.

[48] Kim TM, Yim SH, Lee JS, Kwon MS, Ryu JW (2005). Genome-wide screening of genomic alterations and their clinicopathologic implications in non-small cell lung cancers.. Clin Cancer Res.

[49] Yakut T, Schulten HJ, Demir A, Frank D, Danner B (2006). Assessment of molecular events in squamous and non-squamous cell lung carcinoma.. Lung Cancer.

[50] Candi E, Dinsdale D, Rufini A, Salomoni P, Knight RA (2007). TAp63 and DeltaNp63 in cancer and epidermal development.. Cell Cycle.

[51] Broers JL, Viallet J, Jensen SM, Pass H, Travis WD (1993). Expression of c-myc in progenitor cells of the bronchopulmonary epithelium and in a large number of non-small cell lung cancers.. Am J Respir Cell Mol Biol.

[52] Schraml P, Kononen J, Bubendorf L, Moch H, Bissig H (1999). Tissue microarrays for gene amplification surveys in many different tumor types.. Clin Cancer Res.

[53] Bergh JC (1990). Gene amplification in human lung cancer. The myc family genes and other proto-oncogenes and growth factor genes.. Am Rev Respir Dis.

[54] Prudkin L, Wistuba (2006). Epidermal growth factor receptor abnormalities in lung cancer. Pathogenetic and clinical implications.. Ann Diagn Pathol.

[55] Kang JU, Koo SH, Kwon KC, Park JW, Kim JM (2008). Gain at chromosomal region 5p15.33, containing TERT, is the most frequent genetic event in early stages of non-small cell lung cancer.. Cancer Genet Cytogenet.

[56] Garnis C, Davies JJ, Buys TP, Tsao MS, MacAulay C (2005). Chromosome 5p aberrations are early events in lung cancer: implication of glial cell line-derived neurotrophic factor in disease progression.. Oncogene.

